# moreThanANOVA: A user-friendly Shiny/R application for exploring and comparing data with interactive visualization

**DOI:** 10.1371/journal.pone.0271185

**Published:** 2022-07-08

**Authors:** Wanyanhan Jiang, Han Chen, Lian Yang, Xiaoqi Pan

**Affiliations:** 1 School of Public Health, Chengdu University of Traditional Chinese Medicine, Chengdu, Sichuan Province, China; 2 State Key Laboratory of Grassland and Agro-ecosystem, College of Ecology, Lanzhou University, Lanzhou, Gansu Province, China; Chuo University, JAPAN

## Abstract

In the case of comparing means of various groups, data exploration and comparison for affecting factors or relative indices would be involved. This process is not only complex requiring extensive statistical knowledge and methods, but also challenging for the complex installation of existing tools for users who lack of statistical knowledge and coding experience. Like, the normal distribution and equal variance are crucial premises of parametric statistical analysis. But some studies reported that associated data from various industries violated the normal distribution and equal variance, parametric analysis still involved leading to invalid results. This is owing to that the normal distribution tests and homogeneity of variance test for different variables are time-cost and error-prone, posing an urgent need for an automatic and user-friendly analysis application, not only integrating normal distribution tests and homogeneity of variance test, but also associated the following statistical analysis. To address this, we developed a Shiny/R application, moreThanANOVA, which is an interactive, user-friendly, open-source and cloud-based visualization application to achieve automatic distribution tests, and correlative significance tests, then customize post-hoc analysis based on the considerations to the trade-off of Type I and Type II errors (deployed at https://hanchen.shinyapps.io/moreThanANOVA/). moreThanANOVA enables novice users to perform their complex statistical analyses quickly and credibly with interactive visualization and download publication-ready graphs for further analysis.

## Introduction

When comparing means of various groups, data exploration and comparison associated with various statistical knowledge and methods would be involved [1–3]. Like, most significant statistical analysis would require to assessing the assumption of normality, especially in parametric statistical analysis, the normal distribution being an underlying premise. This owing to the central limit theorem (CLT), when independent random variables are added, their properly normalized sum tends toward a normal distribution. CLT is the theoretical foundation for the premise of parametric statistical analysis. Interestingly, according to some studies [4–6], the associated real data covering various industries presented high levels of deviations and skewed distributions, and parametric statistical analysis still involved tendering unreliable conclusions. When data violate the normal distribution, data transformation (like z-scores or generalized logit) often is applied to comply the normal distribution [7]. If the transformed data still violate the assumption of normality or homogeneity of variance, non-parametric tests or other test methods should be concerned to reflect the data distributions, instead of parametric tests. Hence, the normality test and homogeneity of variance test procedures are the premises of significance tests. When the normality or homogeneity of variance assumption is violated and still applied parametric tests, rendering unreliable or invalid results with lower power of a test.

As mentioned above, the assessments of normality and homogeneity of variance for data is crucial, but these processes are iterative and error-prone, so they need to be automated. After the parametric analysis or non-parametric analysis is determined, significance tests and post-hoc analysis would be assessed, which require multiple steps of data processing, substantial statistical knowledge, complex installation in specific computational environments, and experienced coding skills. In addition, there is no uniform test method for the post-hoc tests yet, which is according to the focus of research, mainly controlling Type I or Type II errors, and this could be another step misleading the results of significance analysis.

**Fig 1:**
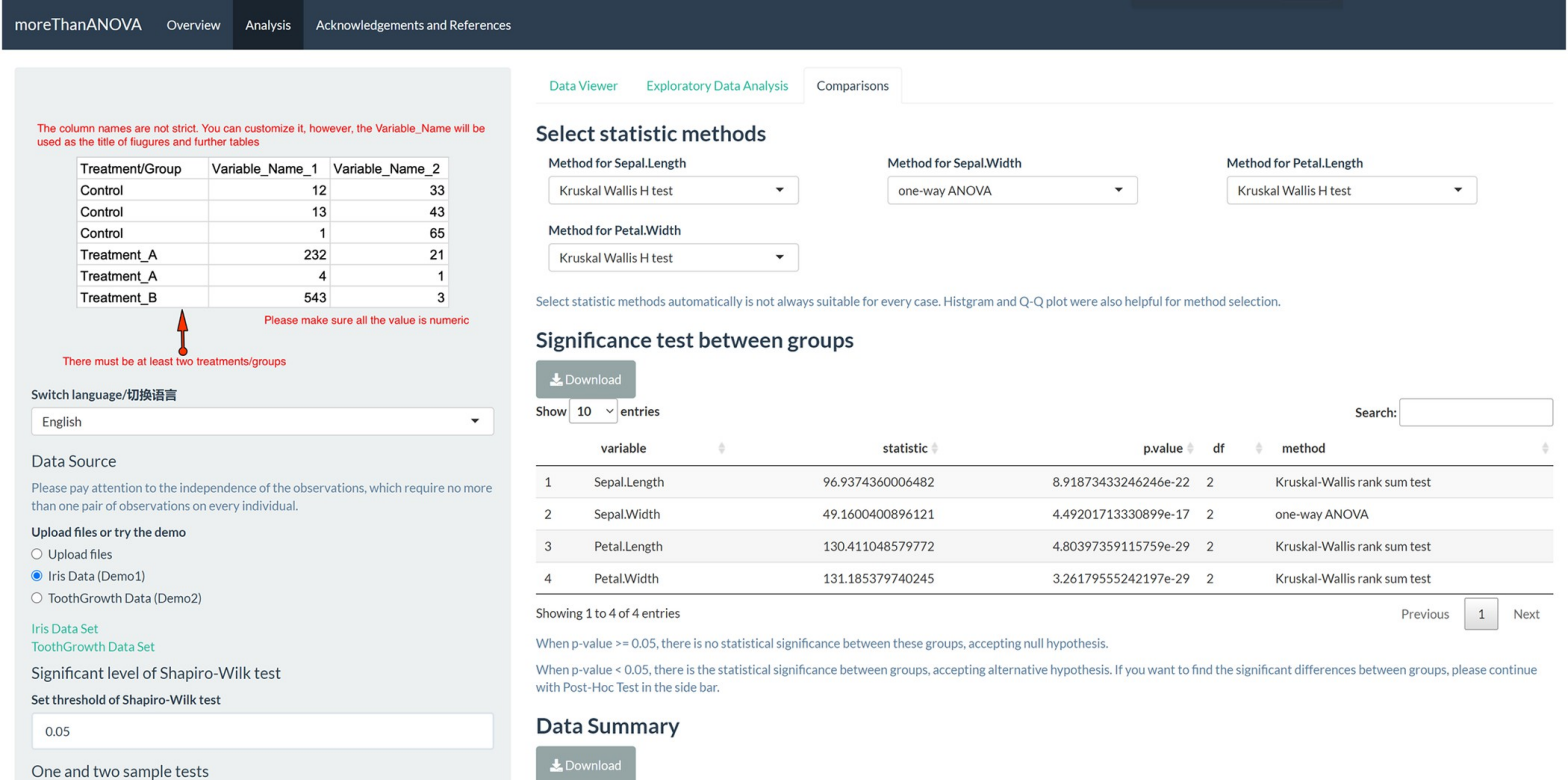
The user interface of Comparisons tab of moreThanANOVA. Reprinted under a CC BY license, with the permission from Han Chen, original copyright 2022.

Visual representations of data are key tools to achieve widespread use of data analytics with visual insight to patterns of the observed data in a simple and easy way. In this case, we developed an R code application, moreThanANOVA, which is an effective, automatic, free, open-source and cloud-based data analysis and visualization application, to quickly and accurately perform the automatic distribution tests and significance tests, and customized post-hoc tests (deployed at https://hanchen.shinyapps.io/moreThanANOVA/). The user interface of moreThanANOVA is displayed in **Fig 1**. This application only requires an input table with each column as a variable and except the first column as treatments or groups labels. Users can highly customize the methods of post-hoc analysis and style of graphics files, then output high-quality figures reaching publication levels, to meet the data exploration and comparison from different industries.

## Statistical methods and stages

The stages of moreThanANOVA are (1) automatic data exploration, data distribution tests, homogeneity of variance test, and data visualization with density plot; (2) based on the data distribution, and the number of groups, automatically determining the methods of significance tests from the Student’s t-test, one-way Analysis of Variance (ANOVA), Wilcoxon Signed Rank test, Wilcoxon Rank Sum test and Kruskal-Wallis H test [8–11]. Besides, users can Monte Carlo Permutation Test in side bar when unknown distribution or small sample size is involved; (3) based on the considerations to the trade-off of Type I and Type II errors, users implementing the methods of post-hoc analysis from the Tukey’s test, Dunn’s test, Bonferroni/Holm test, Hochberg/Hommel test, and data visualization through highly customized box point plot with significance levels [12–15].

### Data distribution tests

The normal distribution and equal variance are an underlying assumption of many significance tests, so we determine the distribution and variance of data through distribution tests and homogeneity of variance test first. In moreThanANOVA, the Shapiro-Wilk test is applied to confirm data are normalized distribution or not, since the Shapiro-Wilk test presents higher test power of normal distribution compared with the Kolmokolov-Smirov test [16–18]. The Levene test is involved in the homogeneity of variance test.

### Significance tests

**Fig 2:**
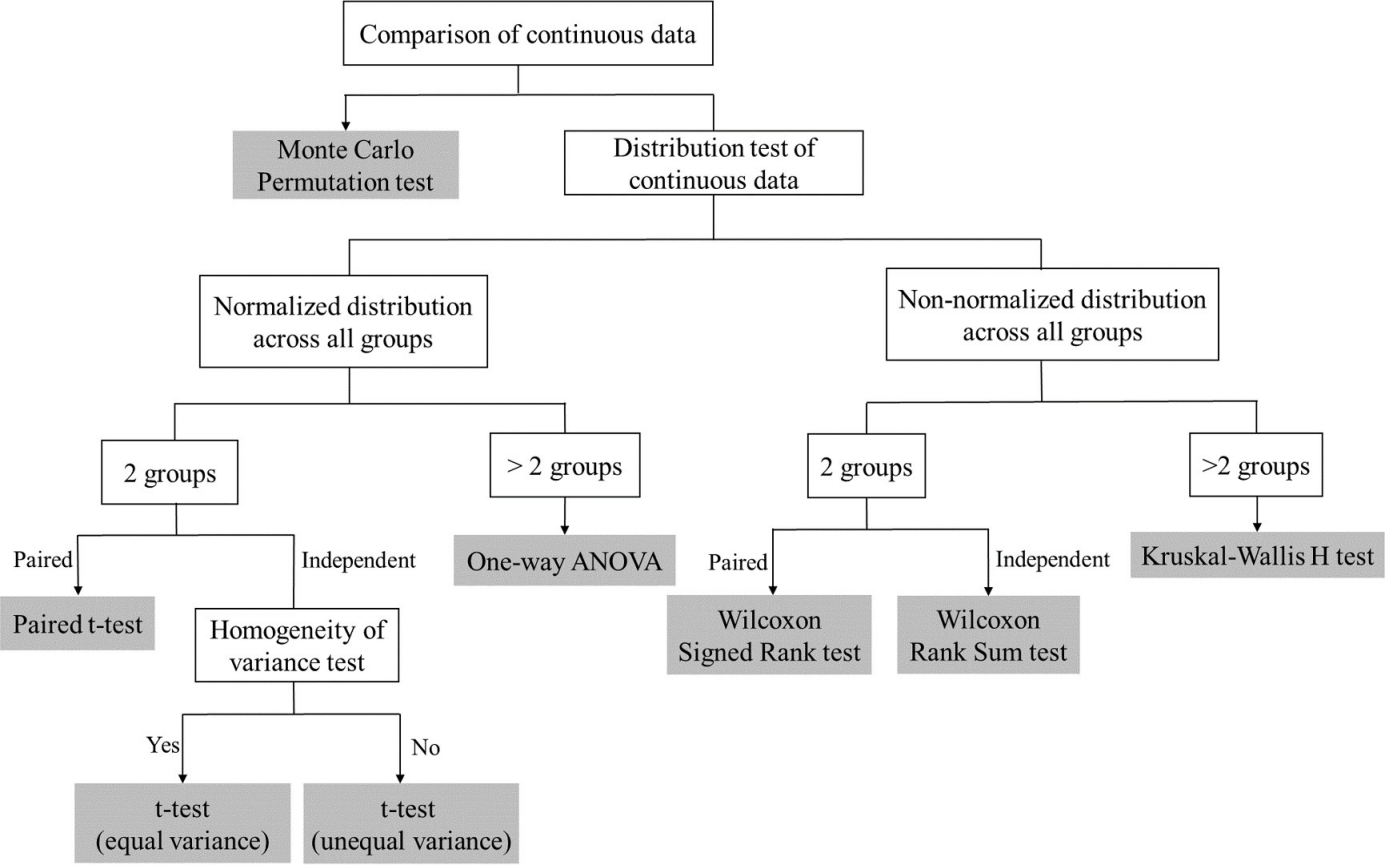
The selection of significance tests based on different scenarios.

A significance test is a method often used to determine if there is any difference between two or more grouped data [19]. A significance test is a statistical hypothesis test, in which the original hypothesis is usually defined as "there are no significant differences between samples". Before performing a significance test on data, the method of significant analysis needs to be selected based on the data distribution and the number of groups, owing to these two factors being in line with various significance tests. If the data are normalized and equal variance, parametric analysis should be adopted to perform significance tests. While data violate the assumption of normality or equal variance, non-parametric tests should be involved at the stage of significance tests. Additionally, benefiting from the computational-intensive theory, it is possible for the data with unknown distribution and a small sample size to deduce more robust statistical results through permutation tests (Monte Carlo permutation test) [20], compared with rank tests. moreThanANOVA covers all the scenarios of parametric tests and non-parametric tests, automatically performing the reliable significance test, which is a user-friendly process. Details are displayed in **Fig 2**.

### Post-hoc tests

Owing to the result of significance tests for multiple data groups, it can only test that if there is any difference between all data groups, without pointing out differences between particular groups, therefore post-hoc analysis is associated to assess the significant differences between particular groups [21]. To date, there has been no uniform solution for post-hoc tests, owing to the considerations to the trade-off of Type I and Type II errors [19]. To achieve its widely application, in moreThanANOVA, users can customize the post-hoc tests from Tukey’s test, Dunn’s test, Bonferroni/Holm test and Hochberg/Hommel test.

## Programming implementation

moreThanANOVA is entirely implemented in R language, whose construction relies on Shiny, a framework to make interactive web applications [22, 23]. Considering the requirements for various statistical analysis, moreThanANOVA involved three statistical analysis steps and panels, namely data input, data analysis, and data output, with details as below.

### Data input

In moreThanANOVA, input data table should be in.csv format using UTF-8 encoding, with each column as a variable and except the first column as treatments or groups labels. The input data table can be uploaded to the application through the Data Source panel of Data Viewer tab. The value of variables should be integer or float, while other data types are not supported. When treated the datasets from same source, the column name should keep in accordance.

The presentation of raw data can be found in the Data Viewer tab, to ensure data read entirely by moreThanANOVA and contents fit the requirements of moreThanANOVA. After the check of input data, the raw data are ready for further analysis.

### Data analysis

In the Data Analysis tab, moreThanANOVA would perform the distribution tests for the different treatments/groups data. As a widely applicable test of distribution tests, the Shapiro-Wilk test is performed by shapiro.test() in stat package. With the significance threshold being set at p value > 0.05 in default, when the results all showing with p value > 0.05, these data are normally distributed with marking as normal, or vice versa labelling non-normal, with the results presenting in a table at Data Distribution panel.

At the Comparison Methods panel of this tab, based on if each variable for different treatments/groups is normally distributed or not and the number of groups, moreThanANOVA would determine the following significant statistical methods automatically, details as follows:

1. When data are divided into only two groups, and each group is normalized, *t*-test would be associated with significance tests [9, 24, 25].
2. When data are divided into more than two groups, and each group is normally distributed, one-way ANOVA is applied to the significance tests [26, 27].
3. When paired data are divided into two groups, and either group violates the normality or equal variance assumption, the significance tests would base on the Wilcoxon Signed Rank test [10, 28].
4. When independent data are divided into two groups, and either group violates the normal or equal variance assumption, the significance tests would base on the Wilcoxon Rank Sum test [10, 29].
5. When data are divided into multiple groups, with none of the groups are normalized or equal variance, the significance test would base on the Kruskal-Wallis H test [11, 19].

Additionally, as mentioned above, to deduce a more robust conclusion, moreThanANOVA adopts Monte Carlo permutation test by default for data that violate the normal distribution, especially the data whose distribution is unknow and with small sample size [20, 30, 31]. In addition, the test method for non-parametric tests panel in the side bar can switch the methods between rank tests and Monte Carlo permutation test, with rank tests by default.

Based on the scenarios mentioned above, moreThanANOVA would automatically determine the methods of significance tests in Comparison Method panel for each variable for different treatments/groups. In the case of all variables are normalized and equal variance, parametric tests would be involved, or non-parametric tests would be associated to insure the significant statistical methods being applied appropriately.

Eventually, based on the distribution of each variable for different treatments or groups, moreThanANOVA presents data linking with visualization approach (density plot and Q-Q plot) to assist in displaying the data distribution [32–34].

### Data output

The data output is presented in Comparisons tab, with significance tests between groups panels listing statistical coefficients, p-values, degrees of freedom and significance test methods.

The Data Summary panel displays commonly used descriptive indicators including Mean, Standard Error (SE), Median, Inter Quartile Range (IQR) and significance levels for post-hoc analysis for each variable, which grouped by different treatments. As mentioned above, based on the hypothesis of CLT, when data violate the normal distribution, median and IQR should represent the characters of data instead of means or SE [35].

For post-hoc analysis, there is not a uniform test method yet, therefore post-hoc analysis should be decided according to the researcher’s focus, considering to the trade-off of Type I and Type II errors. This option can be customized in the side bar of Post-Hoc Test panel.

Box plot and its derivatives are a class of statistical graphs used to describe the variation of grouped data and the display of significance [36–38]. In the Post-Hoc Test panel, moreThanANOVA provides a highly customized and user-friendly interface to set X/Y names and styles, display switches for significant p-values/asterisk, layout, and specifications of box point plot. moreThanANOVA allows users to download the customized figures with publication quality for further analysis.

## Practical case study

The four steps of using moreThanANOVA are (1) uploading files or try the demo at the Data Source panel, (2) clicking the Data Viewer tab to ensure data read entirely by moreThanANOVA, (3) clicking the Exploratory Data Analysis tab to ensure the results of normality test and homogeneity of variance test, in addition with density plot and Q-Q plot, (4) clicking the Comparisons tab to ensure and customize statistic methods, post-hoc test then output customized publication level figures. Besides the basic function, the customized characteristics of setting threshold of Shapiro-Wilk test, the paired or unpaired test, the involve of permutation test, and the method of post-hoc test can be found in the side bar, to perform the customized and easy significance test.

### Binary data

The demo data of binary data is ToothGrowth data (Demo2) in Data Source panel. First, in Data Viewer tab, the original data can be found, with two columns. Secondly, in Exploratory Data Analysis tab, the variable-len is normal and equal variance and *t-* test is suggested. While for the variable-dose is non-normal and unpaired, Wilcoxon Rank Sum test is suggested. Thirdly, in Comparisons tab, the results of Exploratory Data Analysis tab are displayed in Select statistic methods panel. If the users prefer other significance test, they can customize in Select statistic methods panel, only with possible statistical analyses as candidates. Finally, the users can customize their high-quality figures then download them.

### Multivariate data

The demo data of multivariate data is Iris data (Demo1) in Data Source Panel. First, in Data Viewer tab, the original data with four variables are listed. Secondly, in Exploratory Data Analysis tab, the Sepal.Length, Petal.Length and Petal.Width are non-normal and Kruskal-Wallis H test is suggested. Since Sepal.Width is normal and equal variance, one-way ANOVA is involved. Thirdly, in Comparisons tab, the recommended statistic methods are listed in Selected statistical methods panel. Finally, the users can customize and download their high-quality figures.

## Conclusions and outlook

To implement the parametric tests as well as the Wilcoxon/ Kruskal-Wallis test, we used the stats package [39]; to implement the Monte Carlo Permutation test, we used the coin package [40]; to implement post-hoc tests, we used the stats package and the rcompanion package [39, 41].

moreThanANOVA is a lightweight, open-source, cloud-based and user-friendly Shiny/R application to visualize and annotate data exploration and comparison for users with limited statistical knowledge and programming experience, outputting reliable and high-quality results relied on highly customized procedures. The function of this app is like its name (moreThanANOVA), aiming at more than ANOVA with non-parametric tests and Monte Carlo permutation test integrating. moreThanANOVA is a wide range of applications can be applied by users to compare means of groups. Additionally, advanced users can deploy moreThanANOVA on local or public servers to provide on-line moreThanANOVA to other users. The development of moreThanANOVA is still proceeding to add more statistical analysis and visualization tool, which might apply to discrete data.

## References

[1] Sieriebriennikov B, Ferris H, de Goede RG. NINJA: an automated calculation system for nematode-based biological monitoring. European Journal of Soil Biology. 2014;61:90–3. 10.1016/j.ejsobi.2014.02.004.

[2] Choe EK, Lee B, Zhu H, Riche NH, Baur D. Understanding self-reflection: how people reflect on personal data through visual data exploration. Proceedings of the 11th EAI International Conference on Pervasive Computing Technologies for Healthcare. 2017:173–82. 10.1145/3154862.3154881.

[3] De Stefani L, Spiegelberg LF, Kraska T, Upfal E. VizRec: A framework for secure data exploration via visual representation. arXiv preprint arXiv:181100602. 2018. 10.1109/dsaa.2019.00039.

[4] Harvey CR, Siddique A. Conditional skewness in asset pricing tests. The Journal of finance. 2000;55(3):1263–95. 10.1111/0022-1082.00247.

[5] Kobayashi K. Analysis of quantitative data obtained from toxicity studies showing non-normal distribution. The Journal of Toxicological Sciences. 2005;30(2):127–34. doi: 10.2131/jts.30.127 15928460

[6] van der Linden WJ. A lognormal model for response times on test items. Journal of Educational and Behavioral Statistics. 2006;31(2):181–204. 10.3102/10769986031002181.

[7] Lin LI-K, Vonesh EF. An empirical nonlinear data-fitting approach for transforming data to normality. The American Statistician. 1989;43(4):237–43. 10.2307/2685370.

[8] Barnard G. Discussion on the spectral analysis of point process (by MS Bartlett). Journal of the Royal Statistical Society B. 1963;25:294. 10.1111/j.2517-6161.1963.tb00508.x.

[9] Fisher RA. Student. Annals of Eugenics. 1939;9(1):1–9. 10.1111/j.1469-1809.1939.tb02192.x.

[10] Wilcoxon F. Individual comparisons by ranking methods. Biometrics Bulletin. 1945;1(6):80–3. 10.1007/978-1-4612-4380-9_16.

[11] Kruskal WH, Wallis WA. Use of ranks in one-criterion variance analysis. Journal of the American statistical Association. 1952;47(260):583–621. 10.1080/01621459.1952.10483441.

[12] Dunn OJ. Multiple comparisons among means. Journal of the American statistical association. 1961;56(293):52–64. 10.1080/01621459.1961.10482090.

[13] Kramer CY. Extension of multiple range tests to group means with unequal numbers of replications. Biometrics. 1956;12(3):307–10. 10.2307/3001469.

[14] Holm S. A simple sequentially rejective multiple test procedure. Scandinavian journal of statistics. 1979:65–70. Available from: https://www.jstor.org/stable/4615733?seq=1.

[15] Benjamini Y, Hochberg Y. Controlling the false discovery rate: a practical and powerful approach to multiple testing. Journal of the Royal statistical society: series B (Methodological). 1995;57(1):289–300. 10.1111/j.2517-6161.1995.tb02031.x.

[16] Shapiro SS, Wilk MB. An analysis of variance test for normality (complete samples). Biometrika. 1965;52(3/4):591–611. 10.1093/biomet/52.3-4.591.

[17] Razali NM, Wah YB. Power comparisons of shapiro-wilk, kolmogorov-smirnov, lilliefors and anderson-darling tests. Journal of statistical modeling and analytics. 2011;2(1):21–33. Available from: https://www.nbi.dk/~petersen/Teaching/Stat2017/Power_Comparisons_of_Shapiro-Wilk_Kolmogorov-Smirn.pdf.

[18] Kolmogorov A. Sulla determinazione empirica di una lgge di distribuzione. Inst Ital Attuari, Giorn. 1933;4:83–91.

[19] McDonald JH. Handbook of biological statistics: sparky house publishing Baltimore, MD; 2009. Available from: http://www.uni-koeln.de/math-nat-fak/genetik/groups/Langer/HandbookBioStatSecond.pdf.

[20] Boik RJ. The Fisher‐Pitman permutation test: A non‐robust alternative to the normal theory F test when variances are heterogeneous. British Journal of Mathematical and Statistical Psychology. 1987;40(1):26–42. 10.1111/j.2044-8317.1987.tb00865.x.

[21] Scheffé H. A method for judging all contrasts in the analysis of variance. Biometrika. 1953;40(1–2):87–110. doi: 10.1093/biomet/40.1-2.87

[22] Chang W, Cheng J, Allaire J, Xie Y, McPherson J. shiny: Web Application Framework for R. R package version 1.4.0.2. https://CRAN.R-project.org/package=shiny. 2020.

[23] Chang W. shinythemes: Themes for Shiny. R package version 1.1.2. https://CRAN.R-project.org/package=shinythemes. 2018.

[24] Box JF. Guinness, gosset, fisher, and small samples. Statistical science. 1987:45–52. 10.1214/ss/1177013437.

[25] Kalpić D, Hlupić N, Lovrić M. Student’s t-Tests. International Encyclopedia of Statistical Science Part 19/Lovrić, Miodrag (ur); Berlin: Springer, 2011; 1559–1563. 2011. 10.1007/978-3-642-04898-2_641.

[26] Fisher RA. XV.—The correlation between relatives on the supposition of Mendelian inheritance. Earth and Environmental Science Transactions of the Royal Society of Edinburgh. 1919;52(2):399–433. 10.1017/s0080456800012163.

[27] Soper H. On the probable error of the correlation coefficient to a second approximation. Biometrika. 1913;9(1/2):91–115. 10.3102/10769986017004315.

[28] Rey D, Neuhäuser M. Wilcoxon-Signed-Rank Test. International Encyclopedia of Statistical Science. 2011:1658–9. 10.1007/978-3-642-04898-2_616.

[29] Haynes W. Wilcoxon rank sum test. Encyclopedia of systems biology. 2013:2354–5. 10.1007/978-1-4419-9863-7_1185.

[30] Harwell MR, Rubinstein EN, Hayes WS, Olds CC. Summarizing Monte Carlo results in methodological research: The one-and two-factor fixed effects ANOVA cases. Journal of educational statistics. 1992;17(4):315–39. 10.3102/10769986017004315.

[31] Metropolis N, Ulam S. The monte carlo method. Journal of the American statistical association. 1949;44(247):335–41. doi: 10.1080/01621459.1949.10483310 18139350

[32] Chang W. R graphics cookbook: practical recipes for visualizing data: O’Reilly Media; 2018. Available from: https://books.google.com.hk/books?hl=zh-CN&lr=&id=T950DwAAQBAJ&oi=fnd&pg=PP1&dq=graphics+cookbook:+practical+recipes+for+visualizing+data:+O%27Reilly+Media%3B+&ots=xL-Bn3AA-j&sig=_F6SnjlMYZN2FMco_NGnmF1UnpE&redir_esc=y&hl=zh-CN&sourceid=cndr#v=onepage&q=graphics%20cookbook%3A%20practical%20recipes%20for%20visualizing%20data%3A%20O’Reilly%20Media%3B&f=false.

[33] Trosset MW. An introduction to statistical inference and its applications with R: CRC Press; 2009. 10.1201/9781584889489.

[34] Wickham H, Grolemund G. R for data science: import, tidy, transform, visualize, and model data: O’Reilly Media, Inc.; 2016. Available from: https://batrachos.com/sites/default/files/pictures/Books/Wickham_Grolemund_2017_R%20for%20Data%20Science.pdf.

[35] Kallenberg O. Foundations of modern probability: Springer Science & Business Media; 2006. Available from: https://books.google.com.hk/books?hl=zh-CN&lr=&id=uL7UBwAAQBAJ&oi=fnd&pg=PA1&dq=Kallenberg+O.+Foundations+of+modern+probability:+Springer+Science+%26+Business+Media%3B+2006.&ots=ijlZjcD01m&sig=oXNC79EMPICg_JosCIe_GMLLl4E&redir_esc=y#v=onepage&q=Kallenberg%20O.%20Foundations%20of%20modern%20probability%3A%20Springer%20Science%20%26%20Business%20Media%3B%202006.&f=false.

[36] McGill R, Tukey JW, Larsen WA. Variations of box plots. The American Statistician. 1978;32(1):12–6. 10.1080/00031305.1978.10479236.

[37] Spear ME. Charting statistics. 1952. Spear ME. Charting statistics. 1952.

[38] Wickham H, Stryjewski L. 40 years of boxplots. Am Statistician. 2011. Available from: https://vita.had.co.nz/papers/boxplots.pdf.

[39] Bolar K. STAT: Interactive document for working with basic statistial analysis. R package version 0.1.0. https://CRAN.R-project.org/package=STAT. 2020.

[40] Hothrn T, Winell H, Hornik K, Wiel MAvd, Zeileis A. coin: Conditonal inference procedures in a permutation test framework. R package version 1.3–1. https://CRAN.R-project.org/package=coin. 2020.

[41] Mangiafico S. rcompanion: Functions to Support Extension Education Program Evaluation. R package version 2.3.25. https://CRAN.R-project.org/package=rcompanion. 2020.

